# The potential GHGs reduction of co-processing aviation biofuel in life cycle

**DOI:** 10.1186/s40643-023-00674-z

**Published:** 2023-08-30

**Authors:** Ziyu Liu, Xiaoyi Yang

**Affiliations:** 1 School of Energy and Power Engineering, Energy and Environment International Center, Beihang University, Beijing, 100191 China; 2 School of Aeronautic Science and Engineering, Beihang University, Beijing, 100191 China

**Keywords:** Co-processing, Sustainable aviation biofuel, Life cycle, Alternative fuel

## Abstract

The challenge of drop-in jet biofuel should couple the reduction of GHGs emission in whole life cycle with economic competitiveness and achieving performance without reducing performance of engine and aircraft. Co-processing was recognized a promising solution due to availability of existing refining infrastructure and facilities. Based on the LCA approach, the quantitative LCA assessment model (AF-3E) has been established for discovering potential GHGs reduction by co-processing. Typical representatives of oily feedstock, including used cooking oil, soybean, rapeseed, peanut, corn oil, Xanthoceras sorbifolia, jatropha and algae, were compared co-processing with HEFA-SPK blend on GHGs and energy consumption in the whole life. Computational framework is integrated into 3 sub-models and 4 modules, which include feedstocks model, fuel model, flight model and electricity module, hydrogen module, methanol module, hexane module. In flight model, the emissions were investigated at LTO condition and cruise condition and transfer to six types of typical aircraft widely used by similarity criterion. Co-processing achieve less energy consumption and GHGs emission than HEFA-SPK blend, which is attributed to less energy consumption in fuel stage. Used cooking oil conducts 8.17% GHGs reduction in 5% bio-feedstock co-processing and 6.39% in 5% HEFA-SPK jet biofuel blend compared with petroleum-based jet fuel. By sensitivity analysis, the vital factors on GHGs have been extracted in whole life cycle. The purpose of this paper is to discover the advantages and vital factors of co-processing. The results would enhance the interests in both LCA and co-processing for sustainable aviation biofuel.

**Graphical Abstract:**

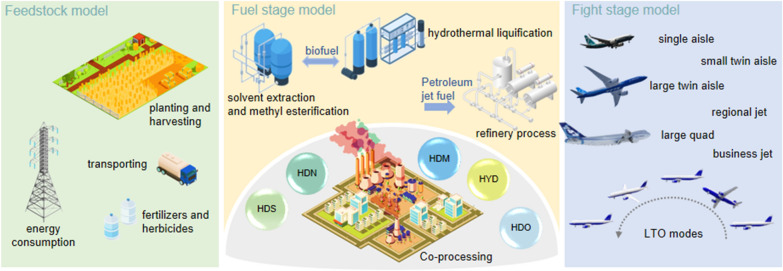

## Highlights

- Establishing LCA model of co-processing with feedstock choice;
- Comparing co-processing with HEFA-SPK blend on GHGs reduction;
- Identifying key factors by global sensitivity analysis.

## Introduction

Green aviation makes an important impact on climate change and clear sky. The diversified development of aviation power requires that aviation energy should comply with the safety and high-speed performance of diversified aircraft, and meet the requirements of green, clean and sustainability. Drop-in jet biofuel is considered a promising available choice without modifications of engine and aircraft and even infrastructure. Drop-in fuel usually is composed of hydrocarbons with same chemical structure as petroleum-derived jet fuel and should be compatible with conventional jet fuel. There are several drop-in fuel processes certified by ASTM, including FT-SPK, HEFA-SPK, HFS-SIP, FT-SPK/A, ATJ-SPK, co-processing, CHJ, and HC-HEFA-SPK (ASTM 2022). Process choice mainly depends on what kind of raw materials. There are mainly three kinds of bio-feedstock: saccharide biomass, lignocellulosic biomass, oil plants and animal fats. Oil plants and animal fats have high-energy lipid as natural oils (free fatty acids or fatty acid esters) with most energy dense storage molecules, and subsequently require relatively less exogenous energy refining into jet fuel. HEFA-SPK and co-processing are both available process for refining drop-in jet fuel. HEFA-SPK jet biofuel is characterized as back-end blend, which should mix below 50% with petroleum jet fuel before use. Co-processing jet fuel is characterized as front-end blend, which is refined by blend bio-feedstock with petroleum.

With the development of HEFA-SPK fuels in technology and environment, economy and investment were becoming a serious obstacle for lipids and oils to aviation biofuel. Petroleum refineries already have a well-developed infrastructure to produce jet fuels, and consequently co-processing would not require additional intensive investments for processing alternative fuel. Therefore, co-processing of fatty acids and fatty acid esters is recognized as promising for jet biofuel to achieve GHGs reduction. Co-processing of biomass-derived feedstocks has already been industrially demonstrated in some cases (Bezergianni et al. 2018). Moreover, biocrude derived from hydrothermal liquefaction (HTL) can be successfully co-processed in a continuous petroleum hydroprocessing unit (Sharma et al. 2021). Elemental analysis revealed that all co-processed fuel were completely deoxygenated. Extensive oxygen and water removal are required to upgrade for fuel-range hydrocarbons, while hydrotreating is one of the most common and cost-effective processes in existing refineries (Wang et al. 2021).

Co-processing usually include hydrotreating, hydrocracking and fractionation as conventional refinery processes. The main challenge of co-processing bio-lipid with petroleum is deoxygenation in existing jet fuel refining units because the formation of water and CO in deoxygenation process could reduce further desulfurization and denitrogenating and even the service life of catalyst in hydrotreating and hydrocracking (van Dyk et al. 2022). The high levels of oxygen in biocrude are a major barrier in co-processing (Goh et al. 2020). According to co-processing HTL biocrude with vacuum gas oil by NiMo/Al_2_O_3_ catalyst in a continuous pilot unit, the addition of distilled biocrude fractions more than 10 vol% could decrease the activity of catalyst in the co-processing (Xing et al. 2019). The potential blend ratio of biocrude or lipid in a petroleum refining scheme remains an open question owing to the unique character of each biocrude (Badoga et al. 2020). Therefore, the current ASTM standard allows less than 5% bio-oil to blend with conventional refinery industry.

For the candidates derived from petroleum refinery steam for co-processing with bio-based feedstocks, the suitable applications include straight run gas oil with used cooking oil (Bezergianni et al. 2012; Sági et al. 2016), straight run diesel with palm oil and soybean oil (Watkins et al. 2008), heavy vacuum gas oil with canola Oil (Chen et al. 2013). The important control condition includes reaction pressure, reaction temperature, space velocity and hydrogen-to-oil ratio, and catalyst. In the co-processing of vegetable oils with petroleum, temperatures above 340 °C could favor conversion efficiency and organic liquid yields but temperatures should be lower than 340 °C due to hydrocracking reactions of hydrogenation and aromatic ring opening reactions (Al-Sabawi and Chen 2012). Hydroprocessing consumes hydrogen, which is obviously related with the type of feedstock characteristics. For catalyst, no catalyst deactivation was observed with a 5% addition in a straight run vacuum distillate with a NiMo catalyst (Tiwari et al. 2011). The current researches confirmed practicability of co-processing in economy and technology. Therefore, the potential GHGs reduction of co-processing jet biofuel should be discovered.

The main objective is to evaluate the potential GHGs reduction and to extract key influence parameters in co-processing jet biofuel. In this paper, the quantitative LCA assessment model was established for discovering potential GHGs reduction by co-processing jet biofuel. According to the LCA approach and AF-3E LCA model (Liu et al. 2022, 2023), feedstock blend for co-processing is compared with HEFA-SPK jet fuel blend in energy consumption and GHGs. The impact of key parameters on GHGs emission was evaluated by uncertainty analysis. The results would enhance the interests in both LCA assessment and co-processing for drop-in jet biofuel.

## Methodology

### Goal definition and system boundary

As the objectives are to compare co-processing jet biofuel (feedstock blend) with direct hydrotreating jet biofuel blend with conventional jet fuel (fuel blend) in energy consumption and GHGs, mass flow and energy flow were involved in the models for assessing the total energy consumption (EC) and GHGs. The functional units of the energy consumption (MJ/kg_jet fuel_ or MJ/MJ_jet fuel_) and GHGs emission (g/kg_jet fuel_ or g/MJ_jet fuel_) are involved to compare the potential GHGs reduction. The functional units of energy consumption (MJ/kg_payload_.km_flight range_) and GHGs emission (kg/kg_payload_.km_flight range_) were chosen in flight range. The GHGs including carbon dioxide (CO_2_), methane (CH_4_) and nitrous oxide (N_2_O), which are all calculated as equivalent to 100 years global warming potentials as gCO_2_e/kg, and volatile organic compounds (VOCs), CO, particulate matter (PM) are also involved in GHGs.

**Fig. 1:**
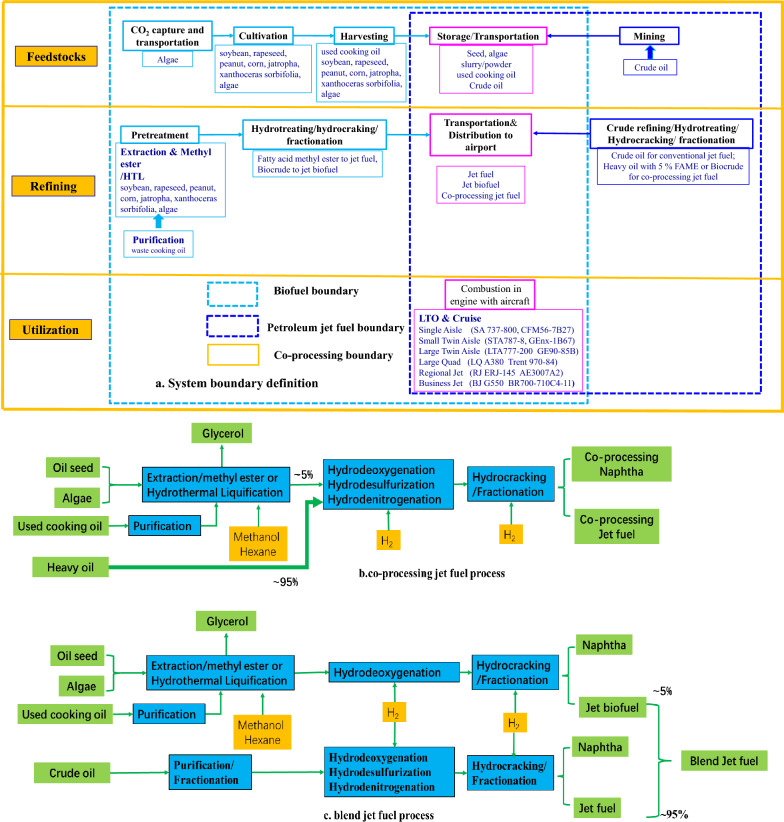
Jet biofuel LCA model

Coupling LCA approach and specific characteristic of jet biofuel, LCA model of co-processing jet biofuel process and blend jet biofuel process are classified as feedstock stage, fuel stage, and flight stage, shown in Fig. 1.

The initial boundary of feedstock was modified with the flexible options for different feedstock. There are 8 bio-feedstocks, including used cooking oil, soybean, rapeseed, peanut, corn, jatropha, Xanthoceras sorbifolia, and algae. Feedstocks selected not only have the ability of economical competitiveness, but also offer the possible production in large-scale. Used cooking oil was mainly derived from soybean, peanut, rapeseed and corn. In recent year, algae, jatropha and Xanthoceras sorbifolia are cultivated in large scale, which are considered as the promising feedstock for biofuel. Initial boundary of algae starts from flue gas capture as CO_2_ source (Liu et al. 2022) while used cooking oil starts from harvesting. Feedstock boundary of soybean, rapeseed, peanut, corn, jatropha, and Xanthoceras sorbifolia include cultivation, harvesting, transportation. For petroleum jet fuel, crude oil start from exploration and recovery process, shown in Fig. 1a.

Co-processing jet biofuel process include bio-feedstock pretreatment module, and co-processing module while blend jet biofuel process includes individual hydrotreating jet biofuel module and individual petroleum jet fuel module. In co-processing process, the precursor of biofuel derived from feedstock pretreatment is mixed with heavy vacuum gas oil derived from petroleum refining for co-hydrotreating, given in Fig. 1b. For blend jet biofuel, precursor of biofuel is individually hydrotreated by two-stage upgrading including hydrotreating and hydrocracking. Jet biofuel is mixed with petroleum jet fuel derived from crude oil as blend jet biofuel, given in Fig. 1c.

In the flight stage, typical civil aircrafts are classified into six types including single aisle, small twin aisle, large twin aisle, large quad, regional jet, and business jet (Greet). Co-processing jet biofuel and blend jet biofuel are both refined to match drop-in jet fuel requirement which do not influence the life time of civil aircrafts with associated engines.

### Computational framework and inventory data

According to system boundary in compliance with functional units, the computational framework is integrated into 3 sub-models and 4 modules, which include feedstocks model, fuel model, flight model and electricity module, hydrogen module, methanol module, hexane module.

In feedstock model, indirect energy consumption is derived from the use of chemical fertilizers and herbicides while direct energy consumption is derived from the use of electricity and power consumption in the process of planting, harvesting and transportation. The impact of nitrogen fertilizers on GHGs release (N_2_O) are involved in GHGs assessment (Liu et al. 2023). Chemical fertilizers and herbicides were involved in cultivation module.

In fuel stage model, there are two pretreatment methods to obtain biofuel precursor. Fatty acid methyl ester (FAME), derived from used cooking oil, soybean, rapeseed, peanut, corn oil, Xanthoceras sorbifolia, jatropha is produced by solvent extraction and methyl esterification, and biocrude (algae) is produced by hydrothermal liquification (HTL). In blend biofuel process, lipids or biocrude is hydrotreated individually into jet biofuel by hydrotreating and hydrocracking, which is considered as HEFA-SPK. The key reactions in hydroprocessing include hydrodenitrogenation and hydrodeoxygenation. Petroleum jet fuel in exploration and recovery process as well as refinery process are based on the current refining technology (Liu and Yang 2020; Liu et al. 2023).

For co-processing biofuel, it includes hydrotreating, hydrocracking and fractionation as conventional refinery processes. Lipids or biocrude blend with heavy vacuum gas oil is upgraded by hydrotreating and hydrocracking into jet biofuel. The key reactions in hydroprocessing include hydrodesulphurization, hydrodenitrogenation, hydrodemetallization, hydrogenation and hydrodeoxygenation.

In flight stage model, the emissions are attributed by distance-weighted average in full envelope including LTO and cruise. Energy consumption and GHGs emission are calculated on per unit load and per unit flight range on the assumption of the maximum flight range. LTO modes include takeoff (thrust 100%, 0.7 min), climb (thrust 85%, 2.2 min), approach (thrust 30%, 4 min), and taxi/idle (thrust 7%, 26 min). By AF-3E model (Liu and Yang 2020; Liu et al. 2023), the electricity module was set based on the consumption of fossil fuel and renewable energy in China, and only algae were involved the effects of CCUS due to algae cultivation by flue gas, given in Table 1. As co-processing was involved in conventional refining process, hydrogen and methanol module were produced by nature gas while hexane was obtained from petroleum.

**Table 1:** Electricity module input and output by AF-3E

Electricity generation mix	China (2019)
Residual oil	0.2%
Natural gas	2.2%
Coal	70.1%
Nuclear power	4.1%
Biomass	1.0%
Hydroelectric	17.2%
Geothermal	0.5%
Wind	3.6%
Solar PV	1.0%
Others	0.10%
Electricity carbon intensity	155 gCO_2_e/MJ
Electricity carbon intensity with CCUS (10% flue gas)	112.2 gCO_2_e/MJ

The cut-off criterion is set at less than 1% on the LCA results as iterative convergence. Carbon sequestration is based on the carbon content in jet biofuel, which comply with the following equation:

$${\text{Carbon}}\;{\text{sequestration}} = - {\text{Biofuel}}\;{\text{blending}}\;{\text{ratio}} \times {\text{fuel}}\;{\text{consumption}}\left( {{\text{kJ}}/{\text{kg}}\;{\text{payload}}.{\text{km}}} \right) \times {\text{carbon }}\left( {\% /{\text{kJ}}_{{{\text{biofuel}}}} } \right) \times {44}/{12}.$$

### Inventory data

The main inventory data were derived from original Chinese government data release and Beihang-AF3E model. Petroleum jet fuel in exploration and recovery process as well as refinery process are based on the original Chinese government data release. LCI in fuel stage are collected from the literature. LCI of petroleum jet fuel in flight stage as the base line were selected from ICAO Aircraft Engine Emission Databank.

The emissions of 5% blend biofuel were investigated in comparison with conventional jet fuel (RP-3) at LTO and cruise condition by ZF850 engine. The emissions were investigated at LTO condition and cruise condition and results transferred to the engine in single aisle by similarity criterion. The further transfer coefficients from single aisle to the other types small twin aisle, large twin aisle, large quad, regional jet, and business jet were estimated coupling characteristics of aircraft with associated engine and average transfer coefficients (Liu et al. 2023). Coupling emission characteristic of different types of engine aircraft with previous research and literature, the emissions of alternative fuels performance were simulated, given in Table 2.

**Table 2:** The inventory in whole life cycle

Feedstock stage	Feedstock	Energy		Material				Further information	
		Electricity kwh/t	Diesel kg/t	N g/kg	P_2_O_5_ g/kg	K_2_O g/kg	Pesticides/herbicide g/kg	Lipid content, %	Release N_2_O kg/kgN
Cultivation	Bean	44.3–77.9	4.4–13.9	2.99	1.5	3.29	0.2–0.3	17%	0.047
	Rapeseed	44.3–77.9	4.4–13.9	29.0	12.5	21.5	0.2–0.3	35%	0.4557
	Peanut	44.3–77.9	4.4–13.9	34	6.5	19.0	0.2–0.3	40%	0.5343
	Corn	44.3–77.9	4.4–13.9	11.8	0.57	0.37	0.2–0.3	8%	0.2719
	Jatropha (Hou et al. 2011; Liu and Qiu 2019)	1.5–1.7	3.2–3.6	19.4	5.4	3.6	0.1–0.3	30%	0.3049
	Xanthoceras sorbifolia (Li et al. 2012; Yao et al. 2013)	1.0–1.3	2.1–2.6	27	7.5	33	0.1–0.3	35%	0.4243
	Algae	2100–2500		16.5	27.5	–	0.2–0.4	40%	0.2593
Harvesting and storage	Bean, rapeseed, peanut, corn	7–22	0.5–2.4						
	algae (powder) (Liu et al. 2020)	55–70	14.8 Heat MJ/kg						
	Algae (powder-solar energy dry) (Liu et al. 2022)	55–72							
	Algae (slurry)	55–68							
Transportation	25–50 km kg/(t km)		0.02					Seed (bean, rapeseed, peanut, corn), algae (powder or slurry), used cooking oil	

Fuel stage		Electricity	Heat (steam)	H_2_ g/g_Jet fuel_	CH_3_OH	Hexane (lost)	Catalyst	Fossil fuel
Pretreatment								
Purification	Used cooking oil (Goh et al. 2020)	0.25 MJ/kg_oil_	0.74 MJ/kg_oil_		0.15 kg/kg_lipid_			
Extraction/methyl ester	Seed for FAME	0.6 MJ/kg_seed_	0.922 MJ/kg_seed_		0.15 kg/kg_lipid_	1.72 g/kg_lipid_		
	Algae (Zhang et al. 2018)	0.65 MJ/kg_algae_	2.73 MJ/kg_algae_		0.15 kg/kg_lipid_			
	HTL biocrude (Tang et al. 2016; Zhang et al. 2016; Sheng et al. 2018)	0.14 MJ/kg_algae_	0.927 MJ/kg_algae_					
Hydrotreating/hydrocracking	FAME (Nie et al. 2014; Wang et al. 2017; Shi et al. 2018)	7.92 MJ/kg_jet fuel_		0.061			1.23 MJ/kg_jet fuel_	
	Biocrude (HTL) (Zhao et al. 2016, 2017)	7.92 MJ/kg_jet fuel_		0.039			1.25 MJ/kg_jet fuel_	
	Co-processing (Bezergianni et al. 2014; Why et al. 2019; Wang et al. 2021)	0.396 MJ/kg_jet fuel_					1.20 MJ/kg_jet fuel_	0.16 MJ/kg_jet fuel_
	Petroleum (Ou et al. 2010; Liu et al. 2023)	0.026 MJ/kg_jet fue_					1.20 MJ/kg_jet fuel_	3.12 MJ/kg_jet fuel_

Flight stage	Test (ZF 850)		Aircraft-single aisle (simulation)		Aircraft-small twin aisle, large twin aisle, large quad (simulation)		Aircraft-regional jet, business jet (simulation)	
5% Blend/RP-3	LTO	Cruise	LTO	Cruise	LTO	Cruise	LTO	Cruise
CH_4_	0.579	0.238	0.579	0.238	0.796	0.327	0.525	0.216
N_2_O	1.0	1.0	1.0	1.0	1.0	1.0	1.0	1.0
CO_2_	0.999	0.999	0.999	0.999	0.999	0.999	0.999	0.999
UHC	0.579	0.238	0.579	0.238	0.796	0.327	0.525	0.216
CO	0.949	1.02	0.949	1.02	1.13	1.22	0.807	0.867
PM	0.209	0.253	0.209	0.253	0.042	0.051	0.057	0.299
SOx	0.949	0.949	0.949	0.949	0.949	0.949	0.949	0.949
NO_x_	1.08	1.09	1.08	1.09	1.05	1.06	1.18	1.19

## Results and discussion

### Feedstocks stage

**Fig. 2:**
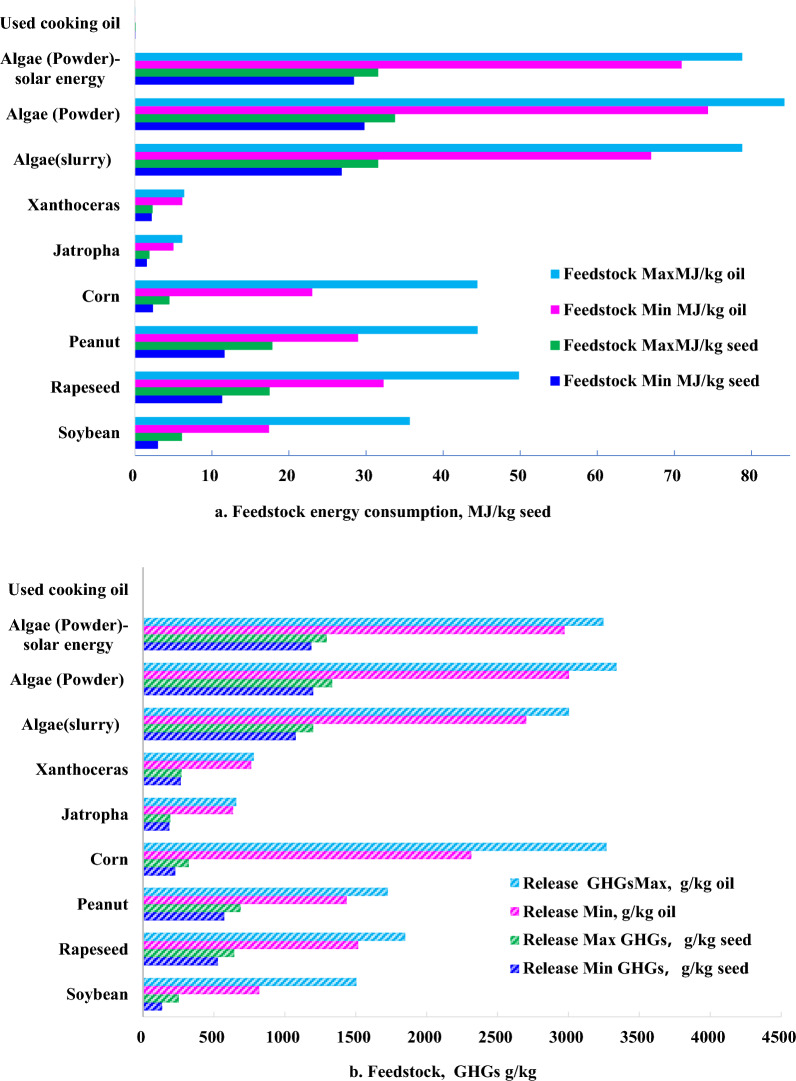
Energy consumption (**a**) and GHGs (**b**) in feedstock stage

Feedstock stage is further classified into cultivation, harvesting, handling and transportation. In comparison in energy consumption, used cooking oil makes an advantage in lowest energy consumption due to no allocation of energy consumption and GHGs emission in cultivation, given in Fig. 2a. In comparison with crop lipid, jatropha and Xanthoceras sorbifolia perform less energy consumption despite based on seed yield and lipid yield due to less energy consumption in cultivation and subsequently less direct energy consumption. The main energy consumptions of jatropha and Xanthoceras sorbifolia are indirect energy consumptions derived from fertilizer, insecticide, herbicide, which occupy above 80% in cultivation stage. Soybean and corn conduct less energy consumption in cultivation stage according to seed yield, which is attributed to less direct energy consumption and less indirect energy consumption. However, according to lipid yield, peanut and rapeseed conduct less energy consumption due to higher lipid content in seed. The highest energy consumption in feedstock is algae in spite of algae slurry and algae powder due to high electricity cost in cultivation.

In GHGs, used cooking oil conducts the least GHGs release due to the lowest energy consumption. According to oil yield (MJ/kg_lipid_), GHGs release in cultivation was ranked as jatropha < Xanthoceras sorbifolia < soybean < peanut < rapeseed < corn < algae while according to seed yield (MJ/kg_seed_), GHGs release in cultivation was ranked as soybean < jatropha < corn < Xanthoceras sorbifolia < rapeseed < peanut < algae, given in Fig. 2b.

### Fuel stage

For co-processing biofuel, the main input of materials in fuel stage contains methanol, hexane, catalyst and hydrogen while the main input of energies are electricity and heat energy. By solvent extraction and methyl esterification, fatty acid methyl ester (FAME) blend with heavy vacuum gas oil is upgraded by hydrotreating and hydrocracking into jet biofuel (co-processing jet fuel). For blend biofuel process, FAME or biocrude is hydrotreated into jet biofuel as HEFA-SPK, which is mixed with petroleum jet fuel derived from petroleum hydrotreating and hydrocracking (blend jet fuel).

**Fig. 3:**
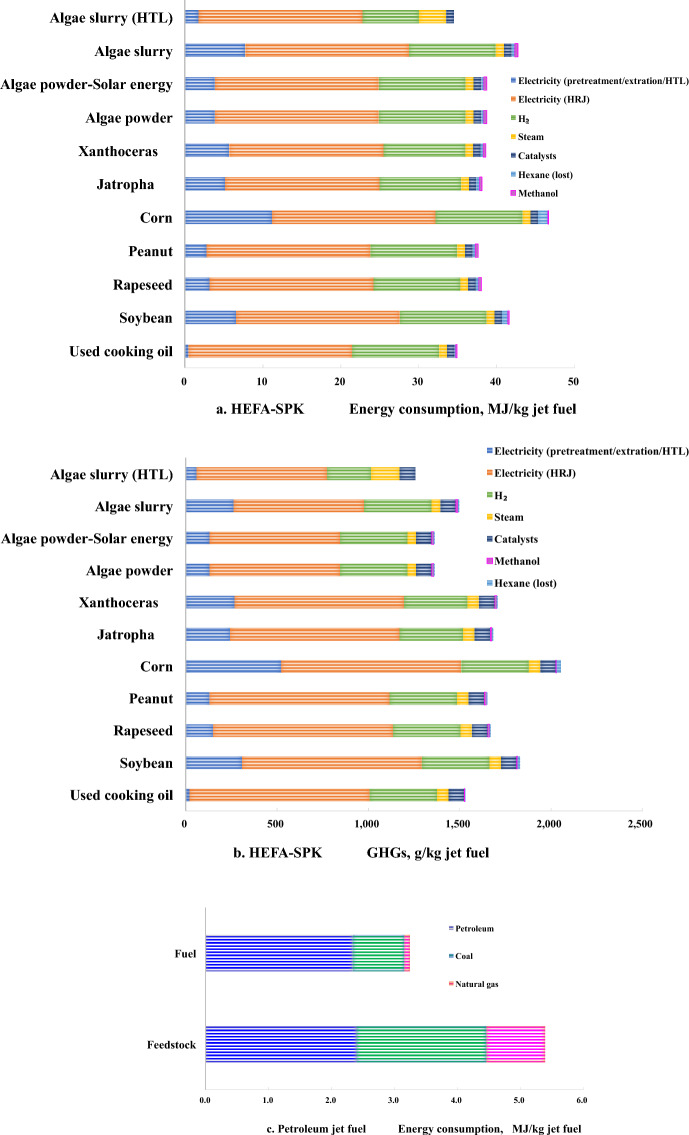

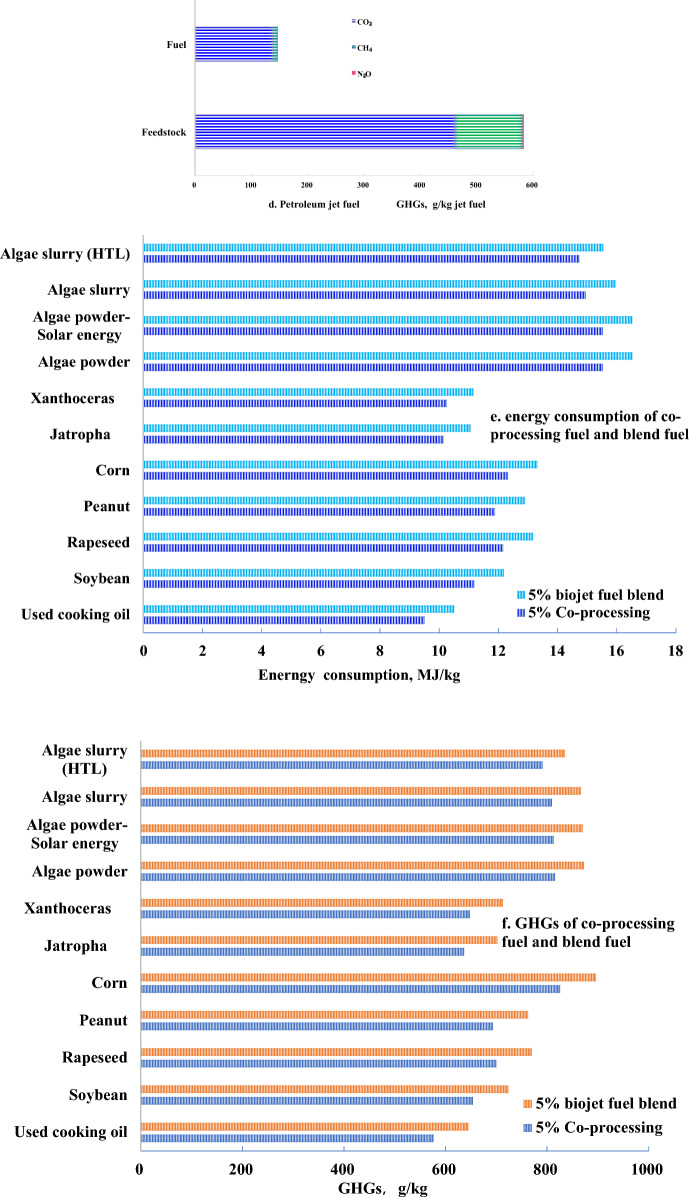
Energy consumption and GHGs in fuel stage. **a** HEFA-SPK energy consumption; **b** HEFA-SPK GHGs; **c** petroleum jet fuel energy consumption; **d** petroleum jet fuel GHGs; **e** Energy consumption of co-processing jet fuel and blend jet fuel; **f** GHGs of co-processing jet fuel and blend jet fuel

Individual bio-feedstock for HEFA-SPK jet biofuel were investigated the total energy consumption and GHGs, given in Fig. 3a, b, and petroleum for jet fuel is given in Fig. 3c, d. As the carbon distribution of fatty acid methyl ester derived from various feedstocks is different, which leads to various HEFA-SPK jet biofuel yield. Used cooking oil in fuel stage conducts the lower GHGs emission and energy consumption due to less energy consumption in pretreatment for hydrotreating precursor. The lipid concentration in feedstock makes an important role to influence the pretreatment efficiency. In spite of any feedstock, electricity occupies the first in GHGs emission in fuel stage, which conduct above 50% GHGs emission, given in Fig. 3b. The second GHGs emission is hydrogen. The integrated of electricity and hydrogen share above 80% GHGs emission in fuel stage for HEFA-SPK. Petroleum jet fuel present less energy consumption and GHGs release in fuel stage than biofuel, given in Fig. 3c, d.

In fuel stage compared with HEFA-SPK blend jet fuel, co-processing jet fuels take both advantage in reduction of energy consumption and GHGs, which were in the range of 5.2–10.7%. Used cooking oil obtained the benefits on 10.7% GHGs reduction and 9.54% energy consumption reduction than HEFA-SPK blend jet fuel, given in Fig. 3e, f, while algae could obtain the benefits on above 5.3% GHGs reduction.

### Flight stage

In flight stage, GHGs emissions conform to a function of fuel consumption and engine efficiency coupling with fuel properties and engine types. Aviation engine are usually designed for optimum engine efficiency at cruise condition and slightly less efficient at LTO cycle. Therefore, jet biofuel blend effects on GHGs emissions present obvious different in LTO cycle and cruise. Therefore, 5% blend jet biofuels were investigated emissions characteristics by ZF850 in comparison with conventional jet fuel at LTO cycle and cruise condition. By integrating emission characteristic with fuel composition, density, C/H ratio and heat value, emissions of co-processing 5% blend have been simulated. In comparison with RP-3, jet biofuel blend comparing with traditional jet fuel has less particulate matter (PM), unborn hydrocarbon (UHC), CH_4_ emission in LTO cycle and in cruise cycle. The results are coincidence with the low sulphur content and low aromatic hydrocarbon content in fuels.

**Fig. 4:**
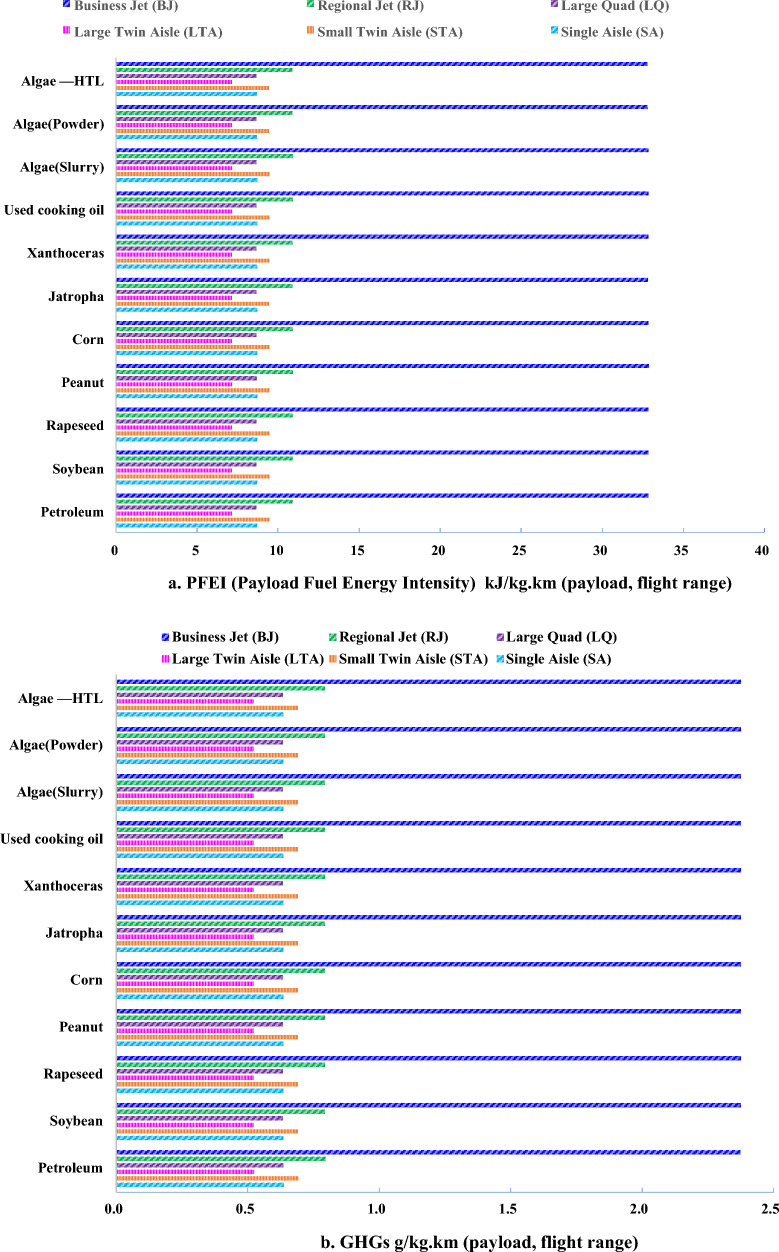
Energy consumption (**a**) and GHGs (**b**) in flight stage

From the view of engine and aircraft effects on GHGs emission in flight stage, 5% jet biofuel blend could reduce GHGs emission slightly respite of LTO cycle or cruise. However, obvious different can be found in six types of engine aircraft while various feedstocks effects on emission conduct less different in flight stage. Large twin aisle aircraft shows the least in energy consumption and GHGs emission than business jet due to high engine efficiency and large payload, given in Fig. 4.

### Whole life cycle assessment and sensitivity analysis

In whole life cycle assessment by AF-3E, GHGs of used cooking oil is 13.3 gCO_2_e/MJ HEFA pathway while petroleum-based jet fuel is 89.9 gCO_2_e/MJ. The default core LCA value in CORSIA for the used cooking oil HEFA pathway is 13.9 gCO_2_e/MJ, which is the average of 14.8 by GREET and 13 gCO_2_e/MJ by E3. The baseline of petroleum-based jet fuel is 89 gCO_2_e/MJ.

**Fig. 5:**
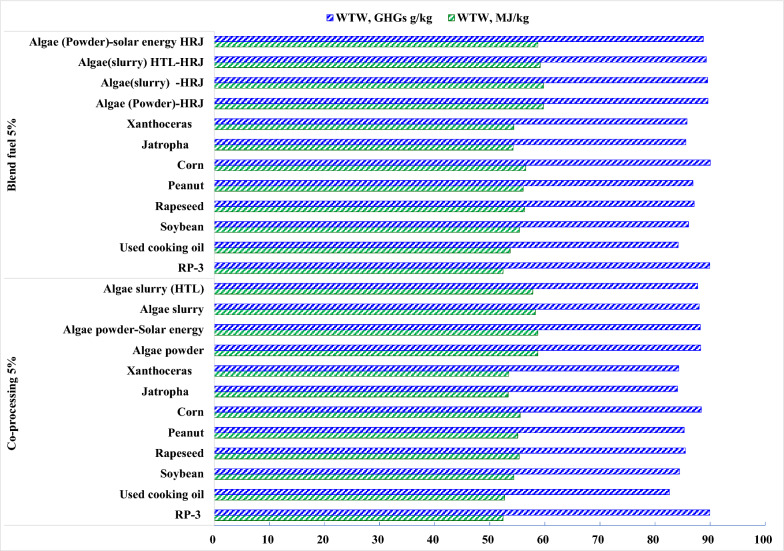
Energy consumption and GHGs in whole life cycle

**Fig. 6:**
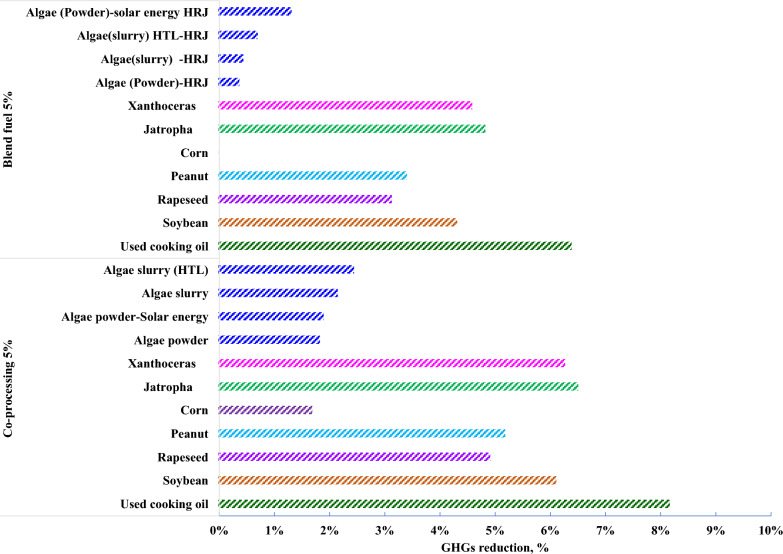
Total GHGs reduction in whole life cycle

GHGs of used cooking oil are 82.67 gCO_2_e/MJ in 5% co-processing and 84.2 gCO_2_e/MJ in 5% HEFA-SPK, given in Fig.5. The benefit is attributed less energy consumption in the fuel stage. Co-processing jet fuels take advantage in reduction of energy consumption and GHGs compared with HEFA-SPK blend jet fuels in spite of any feedstock, given in Fig.6. From the view of feedstock effects, GHGs reductions were achieved by used cooking oil at 8.17%, jatropha at 6.51%, and Xanthoceras sorbifolia at 6.51% in co-processing while by used cooking oil at 6.39%, jatropha at 4.83%, and Xanthoceras sorbifolia at 4.59%.

Global sensitivity analysis is conducted to identify the key factor on the GHGs. The local sensitivity analysis was evaluated in stage level and global sensitivity analysis was evaluated in the whole life cycle. For co-processing, GHGs in flight stage occupied 77–83.8% in whole life cycle while the sum of feedstock stage and fuel stage contributed around 16.2–23%. For 5% HEFA-SPK blend jet fuel, if the lipid content of corn is below 8%, corn cannot achieve GHGs reduction but can achieve GHGs reduction in co-processing jet fuels. The results indicated that the lipid content in feedstock could influence the potential of GHGs reduction in whole life cycle. Moreover, core straw utilization is involved in allocation for heat production or hydrogen production, both of co-processing and HEFA-SPK blend jet fuel blend can further obtain GHGs reduction.

By local sensitivity analysis in feedstock stage, GHGs emission present significantly difference in feedstock stage, lipid content and cultivation energy consumption present obvious effects on GHGs emission. In fuel stage, the sensitive factor is electricity and hydrogen. If CCUS electricity is involved in process, GHGs emission can further reduce. From global sensitivity analysis, engine type and efficiency influence significantly on GHGs reduction.

## Conclusion

Co-processing jet biofuel and HEFA-SPK blend jet fuel blend can both obtain GHGs reduction. In whole life cycle assessment, co-processing jet fuels take advantage in reduction of energy consumption and GHGs compared than HEFA-SPK blend jet fuel, which is attributed to less energy consumption in fuel stage. GHGs reductions were achieved by used cooking oil at 8.17%, jatropha at 6.51%, and Xanthoceras sorbifolia at 6.51% in co-processing while by used cooking oil at 6.39%, jatropha at 4.83%, and Xanthoceras sorbifolia at 4.59%. Feedstock with high lipid content and renewable energy utilization could further reduce GHGs emission for co-processing jet biofuel. According to sensitivity analysis, lipid content and cultivation energy consumption in feedstock stage, electricity and hydrogen in fuel stage, engine type and efficiency in flight stage play vital roles on GHGs reduction.

## Data Availability

The datasets used and/or analyzed during the current study are available from the corresponding author on reasonable request.

## Abbreviations

LCA: Life cycle assessment
LTO: Landing and takeoff
LCI: Life Cycle Inventory
GTL: Gas to liquid
CCUS: Carbon capture, utilization and storage
GHGs: Greenhouse gases
SPK: Synthesized paraffinic kerosine
FT: Fischer–Tropsch
FT-SPK/A: FT synthesized paraffinic kerosine plus aromatics
HEFA: Hydroprocessed esters and fatty acids
HFS-SIP: Hydroprocessed fermented sugars synthesized iso-paraffins
CHJ: Catalytic hydrothermolysis jet
ATJ: Alcohol-to-jet
HC-HEFA: Hydroprocessed hydrocarbons HEFA
FAME: Fatty acid methyl ester
HTL: Hydrothermal liquification
RP-3: Traditional jet fuel
CORSIA: Carbon Offsetting and Reduction Scheme for International Aviation
